# Differential proteomic comparison of breast cancer secretome using a quantitative paired analysis workflow

**DOI:** 10.1186/s12885-019-5547-y

**Published:** 2019-04-18

**Authors:** Giselle Villa Flor Brunoro, Paulo Costa Carvalho, Valmir C. Barbosa, Dante Pagnoncelli, Claudia Vitória De Moura Gallo, Jonas Perales, René Peiman Zahedi, Richard Hemmi Valente, Ana Gisele da Costa Neves-Ferreira

**Affiliations:** 1 Laboratory of Toxinology, Oswaldo Cruz Institute, Fiocruz, Av. Brasil 4365, Manguinhos, Rio de Janeiro 21040-360 Brazil; 2 Laboratory for Proteomics and Protein Engineering, Carlos Chagas Institute, Fiocruz, Rua Prof. Algacyr Munhoz Mader 3775, CIC, Paraná, 81350-010 Brazil; 3 Systems Engineering and Computer Science Program, Federal University of Rio de Janeiro, Caixa Postal 68511, Ilha do Fundão, Rio de Janeiro 21941-972 Brazil; 4 Laboratory of Applied Molecular Biology, Gynecology Department, Fernandes Figueira Institute, Fiocruz, Av. Rui Barbosa 716, Flamengo, Rio de Janeiro 22250-020 Brazil; 5 Laboratory of Molecular Biology of Tumors, Department of Genetics, State University of Rio de Janeiro, Rua São Francisco Xavier 524, Maracanã, Rio de Janeiro 20550-900 Brazil; 6 Leibniz-Institut für Analytische Wissenschaften-ISAS-e.V, Otto-Hahn-Straße 6b, 44227 Dortmund, Germany; 7 Segal Cancer Proteomics Centre, Lady Davis Institute at the Jewish General Hospital, McGill University, 3755 Chemin de la Côte-Sainte-Catherine, Montréal, H3T 1E2 Canada

**Keywords:** Breast cancer, Nipple aspirate fluid, Paired analysis, Proteome, Breast secretion, Liquid biopsy

## Abstract

**Background:**

Worldwide, breast cancer is the main cause of cancer mortality in women. Most cases originate in mammary ductal cells that produce the nipple aspirate fluid (NAF). In cancer patients, this secretome contains proteins associated with the tumor microenvironment. NAF studies are challenging because of inter-individual variability. We introduced a paired-proteomic shotgun strategy that relies on NAF analysis from both breasts of patients with unilateral breast cancer and extended PatternLab for Proteomics software to take advantage of this setup.

**Methods:**

The software is based on a peptide-centric approach and uses the binomial distribution to attribute a probability for each peptide as being linked to the disease; these probabilities are propagated to a final protein *p*-value according to the Stouffer’s Z-score method.

**Results:**

A total of 1227 proteins were identified and quantified, of which 87 were differentially abundant, being mainly involved in glycolysis (Warburg effect) and immune system activation (activated stroma). Additionally, in the estrogen receptor-positive subgroup, proteins related to the regulation of insulin-like growth factor transport and platelet degranulation displayed higher abundance, confirming the presence of a proliferative microenvironment.

**Conclusions:**

We debuted a differential bioinformatics workflow for the proteomic analysis of NAF, validating this secretome as a treasure-trove for studying a paired-organ cancer type.

**Electronic supplementary material:**

The online version of this article (10.1186/s12885-019-5547-y) contains supplementary material, which is available to authorized users.

## Background

Breast cancer is one of the most common human neoplasms, accounting for approximately one quarter of all cancers in females. Invasive breast cancer is the most common carcinoma in women [1, 2]. Most cases arise from epithelial cells of the mammary ductal system. In non-pregnancy and non-lactating periods, these epithelial cells produce a secretion that, when collected, is called the nipple aspirate fluid (NAF) [3]. As a protein-rich breast-proximal fluid closely related to the tumor microenvironment in cancer patients, NAF constitutes a valuable biological sample to study secreted proteins from tumor cells without contamination by other interstitial fluids or cells [4, 5]. Proteomic studies of human body fluids and tissues are challenging, especially due to the high biological variability. Since breast is a “paired” organ, in unilateral breast cancers, the contralateral non-diseased breast from the same individual can be used as an ideal negative control of the cancerous breast [6], ultimately increasing the statistical power.

In 2014, we evaluated for the first time 14 paired NAF samples from seven patients with unilateral breast cancer by PAGE, zymography, and DIGE strategies. Our results have revealed the existence of very distinct proteomic profiles among patients (i.e., individual differences). However, NAF profiles from both breasts of the same woman were very similar in qualitative terms, although important quantitative differences in protein spot intensities could be observed. Patients with less aggressive tumors shared a similar homogeneous profile, with a typical set of proteins identified. In contrast, patients with more aggressive tumors presented very unique profiles (i.e., heterogeneous) [7].

DIGE poses as the state of the art method for sample comparisons by two-dimensional gel electrophoresis. When using this technique, to perform a statistical analysis of spot intensity differences between two conditions (Cy3- and Cy5-labeled), the images are overlaid using the Cy2-labeled internal standard as the reference image. This standard is made up of equal amounts of all samples in the study [8]. Due to the substantial individual heterogeneity found in NAF samples, it was not possible to confidently overlay their gel images, therefore hampering the use of statistical tests for pinpointing differentially abundant candidate markers between the cancer and the control samples. Although the study failed to provide valuable candidate markers, it was fundamental to demonstrate that, even though substantial qualitative individual differences were observed, when comparing NAF samples from both breast within the same patient, the electrophoretic patterns were very similar, regardless of their cancer status [7].

To overcome the limitations imposed by our previous gel-based analytical strategy, a shotgun label-free proteomic approach was applied to further advance the proteomic characterization of NAF samples. However, attempts to use classical data analysis tools (e.g. Proteome Discoverer and Progenesis) [9, 10] were not successful in providing differential results. The significant inter-individual variability of NAF samples confuses chromatogram alignment, which constitutes an important first step of many algorithms. Most importantly, such traditional shotgun proteomic statistical algorithms do not capitalize on the sample pairing. As normalization is not trivial across the patients, applying data analysis strategies that rely on statistically finding differential abundance by considering the average values of each group (cancer vs control) for each protein is simply not applicable for the task at hand; these tools work considerably better for models with lower biological variation, such as cell cultures or mouse models [11, 12].

Taken together, our cumulative experience on various studies made clear that the substantial individual heterogeneity of these clinical samples required further development of proteomic data analysis tools. The pairing of the NAF samples constitutes the core of our strategy and capitalizes on the subtle variations within the same patient. Therefore, we developed an extension to the PatternLab for Proteomics suite that was tailored for the data analysis challenges at hand, which finally enabled us to confidently perform a differential proteomic comparison of breast cancer secretome samples (NAF) from patients with unilateral breast cancer. In summary, here we propose a consistent quantitative analysis workflow for the evaluation of a heterogeneous biological fluid that constitutes a valuable source of information with potential applications in clinical evaluation of breast cancer patients.

## Methods

### Sample collection

NAF samples (10 cancerous and 10 control) were collected from both breasts of 10 patients with biopsy-proven unilateral ductal invasive carcinoma, yielding a total of 20 biological samples, plus three individuals with no positive diagnosis of breast disease on either breast (providing six more biological samples). All samples were collected at the Mastology Service of the Fernandes Figueira Institute (IFF) of Fiocruz or at the Gynecology Ambulatory of Lagoa Federal Hospital (Table 1). Eligibility criteria for all subjects were: a) to be post-menopausal; b) no intake of exogenous hormones during the previous six months; c) no breast surgery or chemotherapy; d) no previous clinical evidence of breast disease or cancer. After obtaining the written informed consent (IFF Research Ethics Committee, license 0083/10) and the clinical and imaging confirmation of the diagnosis status, NAF collection and protein quantification were performed as previously described [7]. Briefly, the breast was gently massaged from the chest wall toward the nipple for 5 min followed by warm compress for equal time. The nipple fluids were then aspirated using breast pumps and the fluid droplets were collected using a 10 μL micropipetter (Gilson, Inc., Middleton, WI, USA). Immediately, the diluted NAF samples (10 times in phosphate buffered saline pH 7.4) were centrifuged at 250 x g for 10 min at 6 °C and the supernatant was collected and stored at − 80 °C. The NAF protein concentrations were determined using the bicinchoninic acid protein assay kit (Sigma-Aldrich, St. Louis, MO, USA).

**Table 1:** Reproductive and tumor characteristics of the ten unilateral breast cancer cases and three individuals without breast disease analyzed

Subject	Age (years)	Birth control use / hormonal replacement^a^	Parity / breastfeeding^b^	Familial breast cancer	Bloom-Richardson Grading^c^	Cancer staging^d^	Immunohistochemistry status		
							Estrogen receptor	Progesterone receptor	Human Epidermal Growth Factor Receptor 2
Patient 1	64	yes / no	1 / yes	no	I	I	Positive	Negative	Negative
Patient 2	73	no / no	6 / yes	no	II	I	Positive	Positive	Negative
Patient 3	53	yes / no	3 / yes	no	II	I	Positive	Positive	Negative
Patient 4	66	yes / no	7 / yes	no	III	IIB	Negative	Negative	Negative
Patient 5	85	no / no	3 / yes	no	II	I	Positive	Positive	Negative
Patient 6	51	no / no	2 / yes	no	II	IIIA	Negative	Negative	Negative
Patient 7	54	no / no	1 / no	no	III	IIB	Negative	Negative	Negative
Patient 8	60	yes / no	3 / yes	no	III	IIB	Negative	Negative	Positive
Patient 9	51	yes / no	3 / yes	no	II	IIIB	Negative	Negative	Positive
Patient 10	72	yes / yes	2 / yes	NDA^e^	II	IIB	Positive	Positive	Negative
Individual 1	59	yes / no	4 / yes	yes	–	–	–	–	–
Individual 2	53	yes / no	3 / yes	no	–	–	–	–	–
Individual 3	61	yes / no	1 / yes	no	–	–	–	–	–

^a^Birth control use/hormonal replacement is defined by whether each woman used or not oral contraceptives/ hormone replacement pills

^b^Breastfeeding is defined by whether or not the woman feed the baby with milk from the breast for at least 1 month

^c^Bloom-Richardson grading at Bloom and Richardson (1957)

^d^Cancer staging classification according to the Brazilian National Institute of Cancer (2004)

^e^NDA refers to No Data Available

### Sample preparation

One hundred micrograms of lyophilized NAF proteins were dissolved in 20 μL of 400 mM ammonium bicarbonate/ 8 M urea followed digestion as described elsewhere [13]. The digested peptide mixture was desalted by using homemade tip columns packed with Poros R2 resin (Applied Biosystems, USA). Samples were finally dried in a vacuum centrifuge [14].

### Mass spectrometry data acquisition

Desalted tryptic peptides were resuspended in 100 μL of 0.1% (*v*/v) trifluoroacetic acid. Samples were then analyzed by nLC-MS/MS using an UltiMate 3000 RSLC system (Dionex, USA) coupled to an Orbitrap Elite mass spectrometer (ThermoFisher Scientific, Germany). Initially, peptides were loaded (normalized TIC values between 5 × 10^8^ – 1 × 10^9^, corresponding to 1–4 μL) with 0.1% TFA at 20 μL/min to a 2-cm long (100 μm i.d.) Acclaim® PepMap100 NanoViper Trap column packed with 5 μm silica particles, 100 Å pore size, followed by separation at 250 nL/min on a 50 cm × 75 μm i.d. Acclaim® PepMap100 NanoViper column, both at 60 °C. Peptides were eluted with a gradient of 3 to 45% of 0.1% (v/v) formic acid and 84% (v/v) acetonitrile over 187 min. The spray voltage was set to 1.8 kV with capillary temperature of 275 °C and no sheath or auxiliary gas flow. Full MS spectra were acquired with 1 microscan on the Orbitrap analyzer at a 60,000 resolution (FWHM at *m/z* 400) with a target AGC value set to 1 × 10^6^. For each survey scan (300 to 1500 *m/z* range), up to 10 most abundant precursor ions were sequentially submitted to CID fragmentation and MS2 analysis in the LTQ using the following parameters: MSn AGC target value of 1 × 10^4^, normalized collision energy of 35%, minimum signal threshold of 2000 counts and dynamic exclusion time of 30 s.

### Data analysis

Peptide-spectrum matching (PSM) was performed using the Comet [15] search engine (version 2016.01), which is embedded in PatternLab for Proteomics (version 4.1, http://patternlabforproteomics.org) [16]. Sequences from *Homo sapiens* were downloaded from UniProtKB/Swiss-Prot (containing target 42,402 entries, on September 17, 2018, http://www.uniprot.org/). The final search database, generated using PatternLab’s Search Database Generator tool, included a reverse decoy for each target sequence plus sequences from 127 common contaminants, such as BSA, keratin, and trypsin. The search parameters applied included: fully tryptic and semi-tryptic peptide candidates with masses between 550 and 5500 Da, up to two missed cleavages, 40 ppm for precursor mass and bins of 1.0005 *m/z* for MS/MS. The modifications were carbamidomethylation of cysteine and oxidation of methionine as fixed and variable, respectively. The validity of the PSMs was assessed using the Search Engine Processor (SEPro) [16]. Identifications were grouped by tryptic status, resulting in two distinct subgroups. For each result, XCorr, DeltaCN, and Comet’s secondary score values were used to generate a Bayesian discriminator. A cutoff score was established to accept a false-discovery rate (FDR) of 1%. A minimum sequence length of 6 amino acid residues was required and the results were further filtered to only accept PSMs with precursor mass error of less than 6 ppm. Proteins identified by only one spectrum (i.e. 1-hit-wonders) having an XCorr below 2.0 were excluded from the identification list. The post-processing filter resulted in a global FDR, at the protein level, of less than 1% and was independent of the tryptic status [17].

### Experimental design and statistical rationale

For breast cancer patients, NAF samples were collected (up to three attempts) in a brief time window between the diagnosis and surgery. Even though proteomic differences in NAF due to the activity of ovarian hormones are believed to be negligible [18–20], we were cautious to only include post-menopausal individuals. Through a workflow of only a few steps, a high-resolution and sensitive nLC-MS/MS analysis [21, 22] was carried out for shotgun evaluation of NAF samples.

PatternLab’s XIC extraction tool was used for obtaining the XICs of peptides confidently identified according to SEPro. The XIC extraction of precursor intensity measurements was performed under a tolerance of 9 ppm and acceptable charge states + 2 and + 3. PatternLab’s XIC Explorer was then used to visually assess the distribution of intensities of the label-free quantitations, label each run as control or disease, and tag which samples were from the same patient for further paired analysis. Additionally, PatternLab’s TFold module was used to demonstrate a standard comparison of mean values between two groups: NAF from diseased breasts versus non-diseased ones.

The .xic file provided by PatternLab served as input to a tool named Paired Analyzer (PA), specifically developed for this study. PA begins by normalizing the XICs from each peptide according to the total ion current from each run. The paired analysis of each unique (i.e. proteotypic) peptide required six or more sequential precursor intensity measurements and a minimum fold change of 1.5. Then, for each peptide, the software extracts a list of values according to one of four possibilities: i) when a peptide’s XIC is obtained from data originating from both breasts, an XIC ratio (cancer:control) is recorded; ii) when an XIC is not obtained from either breast, a “0” (zero) is recorded; iii) when an XIC is obtained only from the diseased sample, a “+” (plus) is recorded; iv) when an XIC is obtained only from the control sample, a “–” (minus) is recorded (Table 2). In what follows, PA relies on a peptide-centric approach to assign a *p*-value to each peptide as being differentially abundant. For this, we follow a paired binomial approach. Our model assumes a 50% chance for a randomly selected peptide to be a success relative to each individual patient for which an XIC was obtained from at least one breast, where success is to be understood as that peptide having a ratio greater than 1 or a “+” for the patient in question. A peptide’s number of successes is the random variable X, and we calculate its p-value as the probability P(X > x), given by a sum of binomials, where x is the number of patients for which success was observed. Thus, for the peptide P_a_ (Table 2), the number of successes (x) is 3, the number of trials (n, number of columns not having 0 as a value) is 5, which yields P(X > 3) = 0.5. For P_b_, x = 2 and *n* = 10, yielding P(X > 2) = 0.99. For P_c_, x = 6 and *n* = 6, yielding P(X > 6) = 0.02. In summary, low *p*-values (e.g. *p* < 0.025) link a peptide to the cancer condition; on the other hand, high values (e.g. *p* > 0.975) would link the aforementioned peptide to the control condition.

**Table 2:** Theoretical example for the peptide-centric approach in the PA module of the PatternLab tool

Peptide^a^	1^b^	2	3	4	5	6	7	8	9	10	*p*-value^c^	Avg Log Fold^d^
Pa	0	+	0	0	0	0.19	0.86	1.37	1.63	0	0.50	−0.36
Pb	0.68	1.15	0.30	0.23	+	–	0.18	0.10	0.57	0.42	0.99	−1.53
Pc	0	3.64	+	1.06	+	0	+	0	8.31	0	0.02	1.67

^a^The first column refers to three distinct peptides (Pa, Pb, and Pc)

^b^Columns labeled as 1 through 10 refer to distinct patients

^c^The column labeled as p-value refers to the p-value as obtained from the binomial distribution

^d^The Avg Log Fold column displays the average logarithm to the base 2 of the values in the corresponding row that are greater than 0

Finally, multiple p-values originating from the peptides mapped to a protein are used to perform a meta-analysis to help determine whether that protein can be considered differentially abundant. This analysis is the determination of Stouffer’s Z-score [23] for the data at hand, denoted by *Z* and given as a function of the various peptide p-values as

$$ Z=\frac{\sum \limits_{i=1}^k{w}_i{Z}_i}{\sqrt{\sum \limits_{i=1}^k{w}_i^2}}. $$

In this expression, *k* is the number of peptides; *Z*_*i*_ = *Φ*^−1^(1 − *p*_*i*_), where *Φ* is the standard normal cumulative distribution and *p*_*i*_ the *i-*th peptide’s p-value; *w*_*i*_ is the square root of the count of individuals in which that peptide was identified.

Average fold-changes were calculated considering the logarithms of the ratios to the base 2, allowing for symmetry in the expression rates of more (positive values, Table 2) and less (negative values) abundant proteins in cancer.

The differentially abundant proteins were categorized in pathways according to the Reactome v60 (https://www.reactome.org/) database. The distribution of those proteins was plotted in a graph from PatternLab’s showing the mean of normalized parent ion intensity abundance factor (NIAF) [24] of each identified protein from the NAF samples of the ten patients.

### Selected reaction monitoring (SRM)

From the differentially abundant list of proteins, 12 of them related to glycolysis, complement cascade and platelet activation pathways were selected for further validation. The spectral library was built from the shotgun analysis described in sections 2.3 and 2.4 and loaded at Skyline software (https://skyline.ms/project/home/software/Skyline/begin.view, version 4.1). A total of 87 transitions were selected for SRM according to peptide uniqueness in the human genome; presence in the spectral library with relatively high intensity of signal; without ragged ends (KK, RR, KR or RK); minimum and maximum size of 8 and 25 aminoacids, respectively; only “y” ion types. Six pairs of samples were prepared as described above and the dessalted tryptic peptide mixtures were quantified by Pierce Quantitative Colorimetric Peptide Assay (ThermoFisher Scientific, USA). A total of 0.5 μg of peptides for each sample spiked in 32 fmol of Pierce Retention Time Calibration Mixture (ThermoFisher Scientific, USA) in 1% formic acid (FA) were loaded to a 2 cm precolumn of 75 μm i.d. with 3 μm silica particles and 100 Å pore size (Acclaim PepMapTM 100, Thermo) in 12 μL of 0,1% (*v*/v) FA and 5% (v/v) acetonitrile in water, using an EASY II (Proxeon, USA). Then, separation was performed at 320 nL/min in a PicoChip column, 75 μm i.d. × 15 μm tip × 10.5 cm of H354 ReproSil-Pur C18-AQ 120 Å (New Objective, USA) using an elution gradient of 5 to 45% of 0.1% (v/v) FA and 5% (v/v) water in acetonitrile over 40 min followed by 45–95% over 10 min. The nLC was coupled to a TSQ Quantiva mass spectrometer (ThermoFisher Scientific, Germany). The spray voltage was set to 2.6 kV with capillary temperature of 280 °C, the 60 min acquisition was done with 2 s cycle time, 0.7 Q1 and Q3 resolution (FWHM 508.2 *m/z*), 1,5 mTorr for collision induced dissociation (CID) fragmentation, and collision energy adjusted according to the theoretical equation of this mass spectrometer. After manual refinement of each transition for each sample, the areas of 48 transitions which refer to 9 proteins were exported from Skyline and imported at Paired Analyzer tool for statistical analysis as described above.

## Results

**Fig. 1:**
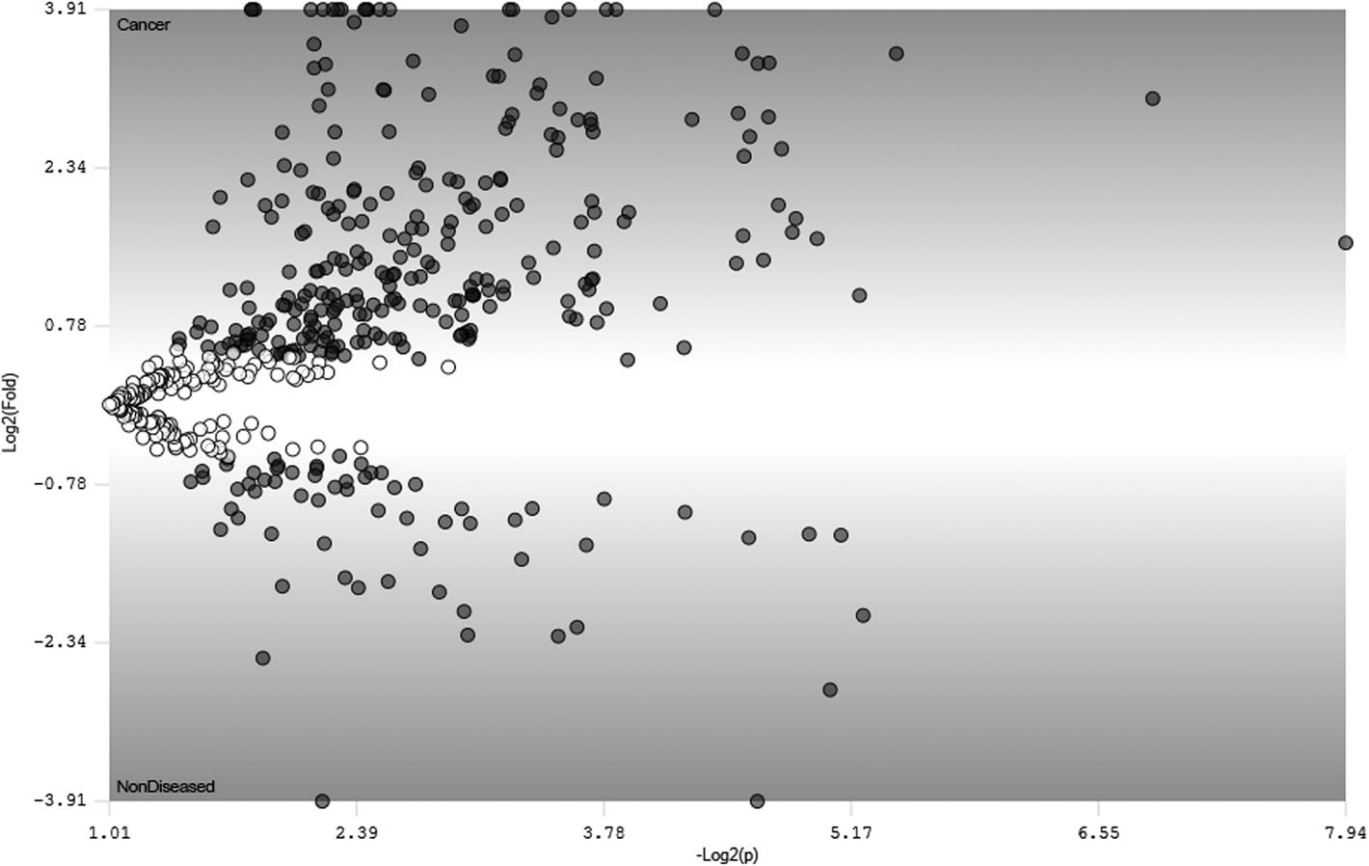
PatternLab’s TFold pairwise analysis of the two biological conditions Each dot represents a protein mapped according to its log2 (fold-change) as the ordinate and its -log2 (t-test *p*-value) as the abscissa. White dots indicate proteins that do not satisfy either the fold-change cutoff or the FDR cutoff α (0.05). Grey dots depict protein entries that satisfy the fold-change cutoff but not FDR α. Dashed dots indicate proteins that satisfy both fold-change and FDR α, but present low fold-changes. Vertical lines filled dots would represent protein entries that satisfy all statistical filters. Since no dashed or vertical lines filled dots are visible, the result interpretation is that no protein was considered differentially abundant between the biological conditions

PatternLab’s TFold comparison of the mean values of protein abundance between the cancerous group versus the non-cancerous one showed no protein as being differentially abundant (Fig. 1).

The shotgun approach disclosed a total of 1227 protein entries (Additional file 1: Table S1), of which 87 proteins (Table 3) were differentially abundant between cancerous and non-diseased breasts from unilateral breast cancer patients, according to our paired statistical approach. From these 87 differentially abundant proteins, all of them were quantified with more than 6 peptides and are included in the Plasma Proteome Database (http://www.plasmaproteomedatabase.org/), proteins except for three immunoglobulins forms (Ig heavy constant gamma 2, Ig kappa variable 3–20, and Ig heavy variable 2–5). Nine differentially abundant proteins were detected in lower levels in NAF samples originating from the cancerous breast (Stouffer *p*-values ≥0.975).

**Table 3:** List of 87 found as differentially abundant after paired comparison of NAF samples from breast cancer patients

Accession number	Stouffer’s p-value^a^	# of unique peptides^b^	Sequence coverage^c^	Protein description^d^
P01023	« 0.05	171	69%	Alpha-2-macroglobulin
P04114	« 0.05	233	48%	Apolipoprotein B-100
P02768	2.89E-15	535	92%	Serum albumin
P02751	5.00E-14	116	51%	Fibronectin
P00450	4.61E-10	138	66%	Ceruloplasmin
P01008	7.85E-10	49	56%	Antithrombin-III
P01024	5.75E-09	293	79%	Complement C3
Q14624	7.30E-08	66	53%	Inter-alpha-trypsin inhibitor heavy chain H4
P02647	7.97E-08	65	79%	Apolipoprotein A-I
P69905	1.77E-06	80	89%	Hemoglobin subunit alpha
P01031	1.86E-06	64	45%	Complement C5
P02787	5.85E-06	233	79%	Serotransferrin
P19823	1.72E-05	40	39%	Inter-alpha-trypsin inhibitor heavy chain H2
P04075	2.11E-05	20	56%	Fructose-bisphosphate aldolase A
P06733	2.772E-05	22	45%	Alpha-enolase
P00751	3.486E-05	62	53%	Complement factor B
P02763	5.823E-05	23	33%	Alpha-1-acid glycoprotein 1
P04217	6.358E-05	39	59%	Alpha-1B-glycoprotein
O43707	8.867E-05	25	32%	Alpha-actinin-4
P02743	8.875E-05	7	28%	Serum amyloid P-component
P63104	0.0001746	17	49%	14–3-3 protein zeta/delta
P08603	0.00018	78	51%	Complement factor H
P62937	0.0001889	7	32%	Peptidyl-prolyl cis-trans isomerase A
P01859	0.00026	41	41%	Immunoglobulin heavy constant gamma 2
P02748	0.0002745	33	57%	Complement component C9
P05109	0.000281	10	65%	Protein S100-A8
P01871	0.000404	76	70%	Immunoglobulin heavy constant mu
P04003	0.00059	49	60%	C4b-binding protein alpha chain
P51884	0.0007103	10	28%	Lumican
P02671	0.0007129	85	53%	Fibrinogen alpha chain
P35579	0.0008537	27	18%	Myosin-9
P00558	0.0009974	13	28%	Phosphoglycerate kinase 1
P62258	0.0012498	9	36%	14–3-3 protein epsilon
P37802	0.0014134	11	42%	Transgelin-2
P01857	0.0014264	44	24%	Immunoglobulin heavy constant gamma 1
P19827	0.0014914	26	33%	Inter-alpha-trypsin inhibitor heavy chain H1
P02652	0.0016813	44	71%	Apolipoprotein A-II
P06744	0.0020099	11	19%	Glucose-6-phosphate isomerase
P31946	0.0020626	11	43%	14–3-3 protein beta/alpha
P60174	0.0023591	21	63%	Triosephosphate isomerase
P02656	0.0023949	7	56%	Apolipoprotein C-III
P01619	0.0024969	13	42%	Immunoglobulin kappa variable 3–20
P13671	0.0031513	34	44%	Complement component C6
P02766	0.0035804	27	69%	Transthyretin
P00338	0.003773	18	45%	L-lactate dehydrogenase A chain
P13639	0.0038429	20	32%	Elongation factor 2
P46940	0.0040271	24	22%	Ras GTPase-activating-like protein IQGAP1
P23528	0.004225	10	40%	Cofilin-1
P00738	0.0043519	57	52%	Haptoglobin
P29401	0.0044629	15	28%	Transketolase
P01019	0.0046976	26	37%	Angiotensinogen
P02679	0.0048135	76	68%	Fibrinogen gamma chain
P43652	0.0048791	33	44%	Afamin
P04406	0.00503	19	55%	Glyceraldehyde-3-phosphate dehydrogenase
Q13228	0.0052836	10	27%	Methanethiol oxidase
P06702	0.005869	16	82%	Protein S100-A9
P05546	0.0059243	21	46%	Heparin cofactor 2
P02794	0.0069928	15	40%	Ferritin heavy chain
O14791	0.0071293	16	36%	Apolipoprotein L1
P04196	0.0102909	37	53%	Histidine-rich glycoprotein
P31151	0.0123024	6	32%	Protein S100-A7
P01817	0.0127189	13	52%	Immunoglobulin heavy variable 2–5
Q99497	0.0143139	6	34%	Protein/nucleic acid deglycase DJ-1
P61981	0.0148811	7	36%	14–3-3 protein gamma
P14618	0.0158523	31	51%	Pyruvate kinase PKM
P01042	0.0159025	35	41%	Kininogen-1
Q96PD5	0.0166293	30	58%	N-acetylmuramoyl-L-alanine amidase
P02792	0.0180461	20	66%	Ferritin light chain
P02750	0.0183273	18	44%	Leucine-rich alpha-2-glycoprotein
P00734	0.0186734	46	54%	Prothrombin
P15311	0.0199674	21	25%	Ezrin
P08758	0.0203289	25	60%	Annexin A5
P32119	0.0204951	13	57%	Peroxiredoxin-2
P02747	0.0210238	6	44%	Complement C1q subcomponent subunit C
P04083	0.0212142	14	41%	Annexin A1
P07900	0.0226553	6	11%	Heat shock protein HSP 90-alpha
P04040	0.022793	33	52%	Catalase
P05543	0.0228794	8	22%	Thyroxine-binding globulin
P08582	0.9849647	41	54%	Melanotransferrin
P19835	0.9915665	74	48%	Bile salt-activated lipase
P08294	0.9923999	19	62%	Extracellular superoxide dismutase [Cu-Zn]
P15144	0.9945681	100	53%	Aminopeptidase N
Q86UK0	0.9954266	16	5%	ATP-binding cassette sub-family A member 12
P12273	0.9980643	104	77%	Prolactin-inducible protein
P22897	0.9986817	81	45%	Macrophage mannose receptor 1
P05090	0.9998501	105	93%	Apolipoprotein D
P02788	1	422	89%	Lactotransferrin

^a^The Stouffer’s p-value obtained from the independent peptide p-values mapping to the respective protein

^b^The # of peptides refers to the number of peptides considered for the calculation of the Stouffer’s p-value

^c^The Sequence coverage was calculated considering all peptides mapping to the respective protein

^d^The Protein description reflects the UniProt description

We also performed a differential analysis between samples from right and left breasts of three women without breast disease (Table 1, individuals 1–3). From the list of 578 statistically evaluated proteins (Additional file 2: Table S2), Ig heavy constant alpha-1 (Stouffer’s p-value = 0.0054), alpha-1-antichymotrypsin (Stouffer’s *p*-value = 0.9888), alpha-1-antitrypsin (Stouffer’s p-value = 0.9943) were pointed as differentially abundant. Since alpha-1-antichymotrypsin and alpha-1-antitrypsin were also found in the comparison between the breasts of cancer patients, they were excluded from the following analyses (Additional file 1: Table S1). Our motivation was to reduce the chance of false positive identifications as, in principle, there should be no reason for having differentially abundant proteins between the NAF samples originating from normal right and left breasts.

**Fig. 2:**
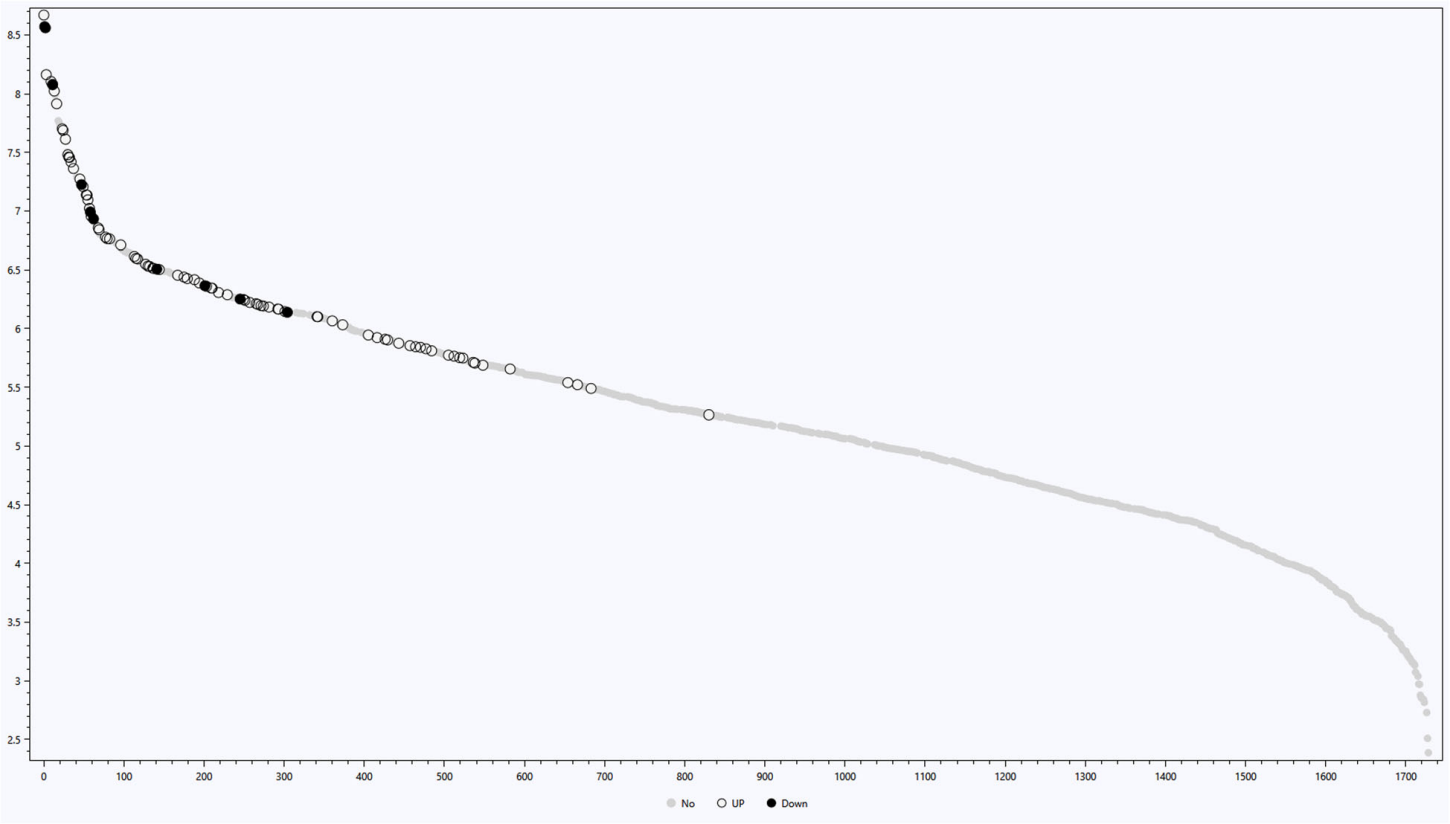
Graph demonstrating the (− 1*10^7) * Log of the average of the normalized ion abundancy factor (NIAF) of all the proteins identified in the NAF For each protein, a number was given as an identifier, and the abscissa is representing these numbers in descending order of abundance. Gray, Black, and White dots represent proteins with no differential abundancy, more abundant in the contralateral non-diseased breasts, and in the breasts with cancer, respectively

Among 87 differentially abundant proteins observed between cancerous and non-diseased paired breasts, it is worth mentioning the frequent identification of proteins associated with the glycolysis pathway, the complement cascade and the platelet activation/degranulation systems (Additional file 3: Table S3). Furthermore, having as reference the average value of NIAF plotted for each protein ordered by abundance, the 87 differentially abundant proteins were among the more abundant ones (Fig. 2).

We also performed an additional differential analysis between NAF samples from a subgroup of patients bearing estrogen receptor-positive tumors (ERpos) (Table 1). From the 873 statistically evaluated proteins (Additional file 4: Table S4), 14 proteins (Table 4) were classified as differentially abundant; 10 of them (alpha-1B-glycoprotein, ceruloplasmin, alpha-2-macroglobulin, serotransferrin, immunoglobulin heavy constant mu, alpha-1-acid glycoprotein 1, ferritin heavy chain, proteinS100-A8, and serum albumin) were also found as differentially abundant in the total set of breast cancer patients. All differentially abundant proteins found in both datasets, ERpos cancer NAF and total cancer NAF, presented the same abundance tendencies. Among the proteins found as more abundant in ERpos samples, representatives of the protein metabolism and the platelet degranulation system were frequently identified (Additional file 3: Table S3).

**Table 4:** List of 14 non-redundant proteins by maximum parsimony criteria found as differentially abundant after paired comparison of NAF samples from ER positive breast cancer patients

Accession number	Stouffer’s p-value^a^	# of peptides^b^	Sequence coverage^c^	Protein description^d^
P02768	1.22E-08	352	87%	Serum albumin
P01023	6.57E-06	113	59%	Alpha-2-macroglobulin
Q6YHK3	0.000399054	32	24%	CD109 antigen
P02787	0.000477172	126	76%	Serotransferrin
P01871	0.002477974	58	70%	Immunoglobulin heavy constant mu
P04217	0.00467967	22	48%	Alpha-1B-glycoprotein
P15086	0.008832761	37	66%	Carboxypeptidase B
P02763	0.012584332	15	33%	Alpha-1-acid glycoprotein 1
P06312	0.014788016	9	36%	Immunoglobulin kappa variable 4–1
P02794	0.02097625	14	40%	Ferritin heavy chain
P00450	0.02154448	94	58%	Ceruloplasmin
Q08380	0.022181763	59	51%	Galectin-3-binding protein
P05109	0.024051217	9	63%	Protein S100-A8
P02788	0.997617412	414	88%	Lactotransferrin

^a^The Stouffer’s p-value obtained from the independent peptide *p*-values mapping to the respective protein

^b^The # of peptides refers to the number of peptides considered for the calculation of the Stouffer’s p-value

^c^The Sequence coverage was calculated considering all peptides mapping to the respective protein

^d^The Protein description reflects the UniProt description

To validate the results obtained from the cancer versus nondiseased comparison, 12 differentially abundant proteins were selected according its overall high MS signal and their presence in the well represented Reactome pathways here described. By Selected Reaction Monitoring (SRM), 4 proteins of glycolysis (pyruvate kinase, glyceraldehyde-3-phosphate dehydrogenase, triosephosphate isomerase, and fructose-bisphosphate aldolase A), 4 proteins of complement cascade (complement C5, complement C3, complement factor B, and complement factor H), and 4 proteins of platelet activation and signaling (alpha-2-macroglobulin, apolipoprotein A-I, fibronectin, and annexin A5). From the initial 87 inicial transitions, 48 were successfully monitored with a CV lower then 15% among replicates, after normalization using global standards. The normalized areas were statistically analyzed by our paired setup and the higher abundance in cancer samples were confirmed to pyruvate kinase, alpha-2-macroglobulin, and complement factor B (*p*-value < 0.05). Although the proteins fructose-bisphosphate aldolase A, complement C5, complement C3, complement factor H, apolipoprotein A-I, and annexin A5 did not reach lower *p*-values, fold changes were corroborated with the higher abundance in cancer samples (Additional file 5: Table S5).

## Discussion

Differential analysis performed with individually paired NAF samples from unilateral breast cancer patients (using the contralateral non-diseased breast sample as negative control) is a powerful strategy for discrimination of which proteins are related to the disease as it helps overcoming the challenge of individual heterogeneity observed between patients [7, 25]. We applied PatternLab’s TFold analysis as a representative of a widely adopted proteomic approach to demonstrate the effectiveness of these methods on datasets with a high biological variation; no proteins were found as differentially abundant. Thus, to quantitatively analyze proteins by label-free shotgun, individual pair-by-pair analysis was performed by the new Paired Analyzer tool of the PatternLab for Proteomics software [26]. As aforementioned, our software performs a quantitative peptide-centric approach that relies on the binomial distribution to attribute a p-value to each peptide as being related to the disease or not. In what follows, these p-values are rolled up to the protein level, converging to the Stouffer’s Z-score via a widely adopted meta-analysis procedure which enables combining independent statistical tests bearing upon the same hypothesis to establish a single score. The tool also allows quickly verifying whether individual peptides belonging to the same protein followed the same trend in differential abundance across the breast cancer NAF samples (Additional file 6: Graphical abstract).

Our shotgun approach revealed proteins presenting higher abundances in breast cancer samples that were previously known as related to cancer progression. Among these proteins are members of the glycolysis pathway, components of the platelet activation/degranulation systems, and proteins associated with the complement cascade [27]. By SRM, at least 2 proteins per pathway corroborated the higher abundance in cancer samples. Increased levels of glycolytic enzymes have been previously related to higher glucose consumption, oncogene activation and loss of function of tumor suppressor genes, promotion of metastasis, angiogenesis stimulation, chemotherapy resistance, and immune evasion [28, 29]. Another known pathway related to tumor progression is the complement cascade that mediates the innate immune system activation, resulting in inflammatory cell and fibroblast recruitment to the tumor microenvironment, which sustains the extracellular matrix remodeling and, consequently, supports cancer progression [30, 31].

In this work we were able to identify 20 representatives of platelet degranulation, activation, signaling or aggregation as more abundant in NAF cancer samples, showing a typically coagulant tumor microenvironment. Several studies report that cancer progression and metastasis (specifically angiogenesis promotion, apoptosis suppression, and extracellular matrix degradation) can be supported by elements of the hemostatic system, such as platelets, coagulation, and fibrinolysis [32]. Therefore, our approach seems to be suitable for more detailed analysis of coagulation cascade proteins, eventually providing further information on its mechanistic relation with breast cancer progression.

Although proteins related to glycolysis and platelet function are commonly found in cancer differential proteomic data [32, 33, 34], their putative roles as moonlighting proteins [35] have been largely overlooked. According to the MultitaskProtDB [36], glucose-6-phosphate isomerase, triosephosphate isomerase, and phosphoglycerate kinase 1, glycolytic proteins which we have found more abundant in the cancer samples, may show moonlighting functions in differentiation/ stimulation of cell migration (as a cytokine or a growth factor) [37], thrombosis/homeostasis [38], and angiogenesis (as a disulphide reductase) [39], respectively. Additionally, the peptidyl-prolyl cis-trans isomerase, an enzyme related to platelet degranulation found more abundant in cancer NAF, presents a proinflammatory cytokine function when located in the extracellular space [40]. Interestingly, our comparative evaluation of the paired breast secretion in unilateral cancer cases showed the presence of these cancer-related proteins extracellularly, which may add important new information to the understanding of the human functional proteome.

Characteristically, ERpos breast tumors are a well differentiated type of cancer and present better treatment response and overall survival [41]. This group of samples showed increased levels of proteins related to the regulation of IGF transport and to the platelet degranulation system. Although some findings about the role of IGF system in breast cancer are conflicting, many components of this system are known to be altered during breast cancer establishment and progression regardless the expression patterns of receptors (ER, PR and HER2) [42]. Overall, proliferation mechanisms which are present in these tumors were observed in this work.

## Conclusions

In NAF cancer samples, the higher abundances of proteins involved in cell-stroma communication, glycolysis (Warburg effect), and immune system activation (to maintain a stimulated stroma) corroborate previous breast cancer data from the literature. Additionally, this paired comparative proteomic strategy of analysis presents valuable information on the mechanisms described above that are known to be related to the disease, even with the inter-individual heterogeneity characteristic of NAF samples. Although, we performed an SRM experiment and confirmed the higher abundance of 3 proteins in cancer samples, further verification/confirmation of higher levels of glycolytic enzymes, complement components, and platelets activators in a larger cohort (> 20 NAF paired samples per cancer subtype, throughout the entire pathways) using the targeted proteomic strategy may contribute to new advances in breast cancer evaluation. Taken together, these results demonstrate that protein analysis of NAF, a clinical sample easily obtained, could compose a pillar in precision medicine, guiding a protein-based prognosis.

## Additional files

Additional file 1: **Table S1.** Result summary of the protein and peptide identifications from the NAF samples from breast cancer patients. a) Protein Report: List of all 1227 proteins statistically evaluated after paired comparisons of NAF samples from breast cancer patients; b) Peptide Report: List of all 1227 proteins and the respective peptides statistically evaluated after paired comparisons of NAF samples from breast cancer patients. (XLSX 1245 kb)

Additional file 2: **Table S2.** Result summary of protein and peptide identifications (NAF samples from control women). a) Protein Report: Complete list of 578 proteins statistically evaluated after paired comparisons of non diseased breast NAF samples from control women; b) Peptide Report: Complete list of 578 proteins and the respective peptides statistically evaluated after paired comparisons of non diseased breast NAF samples from control women. (XLSX 364 kb)

Additional file 3: **Table S3.** Functional analysis of differentially abundant proteins (NAF samples from breast cancer patients). a) Cancer_vs_Nondiseased: Reactome pathways categorization of the proteins found as differentially abundant after paired comparison of NAF samples from breast cancer patients; b) ERpos_vs_Nondiseased: Reactome pathways categorization for differentially abundant proteins according to the paired comparison of NAF samples from ERpos breast cancer patients. (XLSX 26 kb)

Additional file 4: **Table S4.** Summary result of protein and peptide identifications (NAF samples from ERpos breast cancer patients). a) Protein Report: List of all 873 proteins statistically evaluated after paired comparisons of NAF samples from ERpos breast cancer patients; b) Peptide Report: List of all 873 proteins and the respective peptides statistically evaluated according to the paired comparisons of NAF samples from ERpos breast cancer patients. (XLSX 639 kb)

Additional file 5: **Table S5.** Summary result of the proteins monitored by SRM (NAF samples from breast cancer patients). a) Protein Report: List of the 9 proteins monitored by SRM and statistically evaluated after paired comparisons of NAF samples from breast cancer patients; b) Peptide Report: List of the 9 proteins and the respective peptides monitored by SRM and statistically evaluated according to the paired comparisons of NAF samples from breast cancer patients. (XLSX 17 kb)

Additional file 6: Graphical abstract. This study introduced a paired-proteomic shotgun strategy that relies on NAF analysis from both breasts of patients with unilateral breast cancer. The differential analysis of the quantitative data was performed by the “Paired Analyzer”, a newly developed module that works together with the “PatternLab for Proteomics” software. Using a peptide-centric approach, the software applied the binomial distribution to attribute a probability for each peptide as being linked to the disease; these probabilities were propagated to a final protein p-value, according to the Stouffer’s Z-score method. (TIF 195 kb)

## Abbreviations

AGC: automatic gain control
ERpos: estrogen receptor positive
FDR: false discovery rate
FWHM: full width at half maximum
HER-2: human epidermal growth factor receptor 2
LTQ: linear trap quadrupole
NAF: nipple aspirate fluid
nLC: nano liquid chromatography
PA: paired analyzer
PRM: parallel reaction monitoring
PSM: peptide spectrum match
SEPro: Search Engine Processor
SRM: Selected Reaction Monitoring
TIC: total ion current
TNBC: triple negative breast cancer
XIC: extracted ion chromatogram/current

## References

[1] Bray F, Jemal A, Grey N, Ferlay J, Forman D (2012). Global cancer transitions according to the human development index (2008-2030): a population-based study. Lancet Oncol.

[2] Ferlay J, Soerjomataram I, Dikshit R, Eser S, Mathers C, Rebelo M (2015). Cancer incidence and mortality worldwide: sources, methods and major patterns in GLOBOCAN 2012. Int J Cancer J Int Cancer.

[3] Djuric Z, Visscher DW, Heilbrun LK, Chen G, Atkins M, Covington CY (2005). Influence of lactation history on breast nipple aspirate fluid yields and fluid composition. Breast J.

[4] Alexander H, Stegner AL, Wagner-Mann C, Du Bois GC, Alexander S, Sauter ER (2004). Proteomic analysis to identify breast cancer biomarkers in nipple aspirate fluid. Clin Cancer Res Off J Am Assoc Cancer Res.

[5] Varnum SM, Covington CC, Woodbury RL, Petritis K, Kangas LJ, Abdullah MS (2003). Proteomic characterization of nipple aspirate fluid: identification of potential biomarkers of breast cancer. Breast Cancer Res Treat.

[6] Kuerer HM, Coombes KR, Chen J-N, Xiao L, Clarke C, Fritsche H (2004). Association between ductal fluid proteomic expression profiles and the presence of lymph node metastases in women with breast cancer. Surgery..

[7] Brunoro GVF, Ferreira AT da S, Trugilho MR de O, de OTS, Amêndola LCB, Perales J (2014). Potential correlation between tumor aggressiveness and protein expression patterns of nipple aspirate fluid (NAF) revealed by gel-based proteomic analysis. Curr Top Med Chem.

[8] Alban A, David SO, Bjorkesten L, Andersson C, Sloge E, Lewis S (2003). A novel experimental design for comparative two-dimensional gel analysis: two-dimensional difference gel electrophoresis incorporating a pooled internal standard. Proteomics..

[9] Gonzalez-Galarza FF, Lawless C, Hubbard SJ, Fan J, Bessant C, Hermjakob H (2012). A critical appraisal of techniques, software packages, and standards for quantitative proteomic analysis. Omics J Integr Biol.

[10] Qi D, Brownridge P, Xia D, Mackay K, Gonzalez-Galarza FF, Kenyani J (2012). A software toolkit and interface for performing stable isotope labeling and top3 quantification using Progenesis LC-MS. Omics J Integr Biol..

[11] Nie S, McDermott SP, Deol Y, Tan Z, Wicha MS, Lubman DM (2015). A quantitative proteomics analysis of MCF7 breast cancer stem and progenitor cell populations. Proteomics..

[12] Di Luca A, Henry M, Meleady P, O’Connor R (2015). Label-free LC-MS analysis of HER2+ breast cancer cell line response to HER2 inhibitor treatment. Daru J Fac Pharm Tehran Univ Med Sci.

[13] Brunoro GVF, Carvalho PC, Ferreira AT da S, Perales J, Valente RH, de Moura Gallo CV (2015). Proteomic profiling of nipple aspirate fluid (NAF): exploring the complementarity of different peptide fractionation strategies. J Proteome.

[14] Larsen MR, Trelle MB, Thingholm TE, Jensen ON (2006). Analysis of posttranslational modifications of proteins by tandem mass spectrometry. BioTechniques..

[15] Eng JK, Jahan TA, Hoopmann MR (2013). Comet: an open-source MS/MS sequence database search tool. PROTEOMICS..

[16] Carvalho PC, Lima DB, Leprevost FV, Santos MDM, Fischer JSG, Aquino PF (2015). Integrated analysis of shotgun proteomic data with PatternLab for proteomics 4.0. Nat Protoc.

[17] Barboza R, Cociorva D, Xu T, Barbosa VC, Perales J, Valente RH (2011). Can the false-discovery rate be misleading?. Proteomics..

[18] Chatterton RT, Geiger AS, Khan SA, Helenowski IB, Jovanovic BD, Gann PH (2004). Variation in estradiol, estradiol precursors, and estrogen-related products in nipple aspirate fluid from normal premenopausal women. Cancer Epidemiol Biomark Prev Publ Am Assoc Cancer Res Cosponsored Am Soc Prev Oncol.

[19] Huang Y, Nagamani M, Anderson KE, Kurosky A, Haag AM, Grady JJ (2007). A strong association between body fat mass and protein profiles in nipple aspirate fluid of healthy premenopausal non-lactating women. Breast Cancer Res Treat.

[20] Noble J, Dua RS, Locke I, Eeles R, Gui GPH, Isacke CM (2007). Proteomic analysis of nipple aspirate fluid throughout the menstrual cycle in healthy pre-menopausal women. Breast Cancer Res Treat.

[21] Gilar M, Olivova P, Daly AE, Gebler JC (2005). Orthogonality of separation in two-dimensional liquid chromatography. Anal Chem.

[22] Michalski A, Damoc E, Lange O, Denisov E, Nolting D, Müller M (2012). Ultra high resolution linear ion trap Orbitrap mass spectrometer (Orbitrap elite) facilitates top down LC MS/MS and versatile peptide fragmentation modes. Mol Cell Proteomics MCP.

[23] Whitlock MC (2005). Combining probability from independent tests: the weighted Z-method is superior to Fisher’s approach: combining probabilities from many tests. J Evol Biol.

[24] Zhang Y, Wen Z, Washburn MP, Florens L (2015). Improving label-free quantitative proteomics strategies by distributing shared peptides and stabilizing variance. Anal Chem.

[25] Kuerer HM, Goldknopf IL, Fritsche H, Krishnamurthy S, Sheta EA, Hunt KK (2002). Identification of distinct protein expression patterns in bilateral matched pair breast ductal fluid specimens from women with unilateral invasive breast carcinoma. High-throughput biomarker discovery. Cancer..

[26] Carvalho PC, Fischer JSG, Xu T, Yates JR, Barbosa VC. PatternLab: from mass spectra to label-free differential shotgun proteomics. Curr Protoc Bioinforma Ed Board Andreas Baxevanis Al. 2012;Chapter 13:Unit13.19. 10.1002/0471250953.bi1319s40 23255152

[27] Dvorak HF (1986). Tumors: wounds that do not heal. Similarities between tumor stroma generation and wound healing. N Engl J Med.

[28] Jang M, Kim SS, Lee J (2013). Cancer cell metabolism: implications for therapeutic targets. Exp Mol Med.

[29] El Sayed SM, Mohamed WG, Seddik M-AH, Ahmed A-SA, Mahmoud AG, Amer WH (2014). Safety and outcome of treatment of metastatic melanoma using 3-bromopyruvate: a concise literature review and case study. Chin J Cancer.

[30] Byun JS, Gardner K (2013). Wounds that will not heal: pervasive cellular reprogramming in cancer. Am J Pathol.

[31] Mueller MM, Fusenig NE (2004). Friends or foes - bipolar effects of the tumour stroma in cancer. Nat Rev Cancer.

[32] Lal I, Dittus K, Holmes CE (2013). Platelets, coagulation and fibrinolysis in breast cancer progression. Breast Cancer Res BCR.

[33] Martinez-Outschoorn U, Sotgia F, Lisanti MP (2014). Tumor microenvironment and metabolic synergy in breast cancers: critical importance of mitochondrial fuels and function. Semin Oncol.

[34] Calderón-González KG, Valero Rustarazo ML, Labra-Barrios ML, Bazán-Méndez CI, Tavera-Tapia A, Herrera-Aguirre ME (2015). Determination of the protein expression profiles of breast cancer cell lines by quantitative proteomics using iTRAQ labelling and tandem mass spectrometry. J Proteome.

[35] Jeffery CJ (1999). Moonlighting proteins. Trends Biochem Sci.

[36] Hernandez S, Ferragut G, Amela I, Perez-Pons J, Piñol J, Mozo-Villarias A (2014). MultitaskProtDB: a database of multitasking proteins. Nucleic Acids Res.

[37] Chaput M, Claes V, Portetelle D, Cludts I, Cravador A, Burny A (1988). The neurotrophic factor neuroleukin is 90% homologous with phosphohexose isomerase. Nature..

[38] Liu Q-Y, Corjay M, Feuerstein GZ, Nambi P (2006). Identification and characterization of triosephosphate isomerase that specifically interacts with the integrin αIIb cytoplasmic domain. Biochem Pharmacol.

[39] Lay AJ, Jiang XM, Kisker O, Flynn E, Underwood A, Condron R (2000). Phosphoglycerate kinase acts in tumour angiogenesis as a disulphide reductase. Nature..

[40] Jin Z-G, Lungu AO, Xie L, Wang M, Wong C, Berk BC (2004). Cyclophilin a is a proinflammatory cytokine that activates endothelial cells. Arterioscler Thromb Vasc Biol.

[41] Barcellos-Hoff MH (2013). Does microenvironment contribute to the etiology of estrogen receptor-negative breast cancer?. Clin Cancer Res Off J Am Assoc Cancer Res..

[42] Christopoulos PF, Msaouel P, Koutsilieris M (2015). The role of the insulin-like growth factor-1 system in breast cancer. Mol Cancer.

