# Supplementary figures and images for: Differential proteomic comparison of breast cancer secretome using a quantitative paired analysis workflow
Source: BMC Cancer. 2019 Apr 18;19:365. doi: 10.1186/s12885-019-5547-y (PMC6474050; doi:10.1186/s12885-019-5547-y)

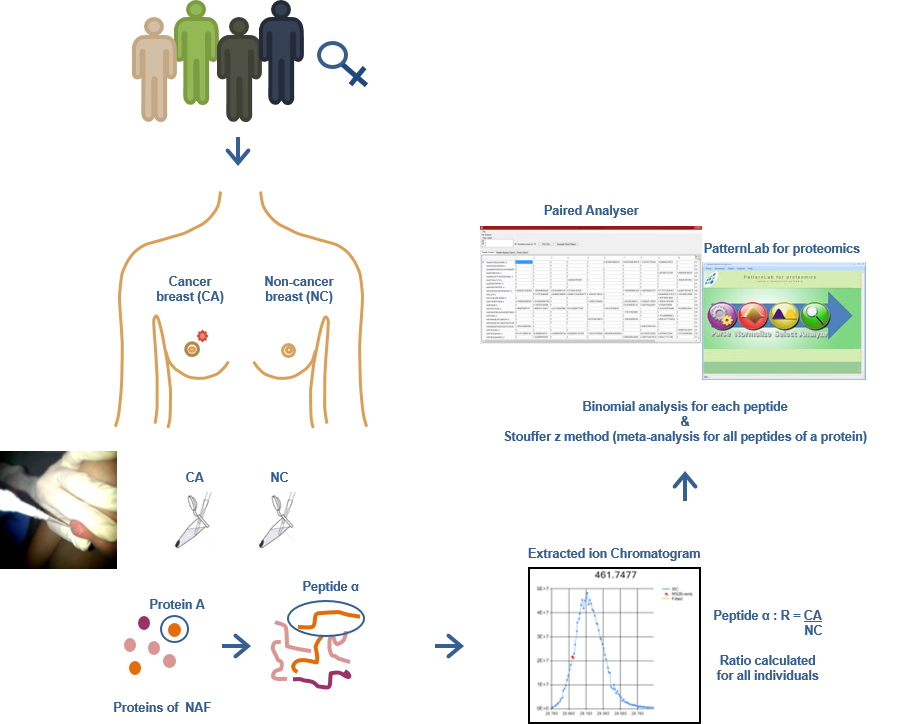

Supplement: Supplementary file 6 — Graphical abstract. This study introduced a paired-proteomic shotgun strategy that relies on NAF analysis from both breasts of patients with unilateral breast cancer. The differential analysis of the quantitative data was performed by the “Paired Analyzer”, a newly developed module that works together with the “PatternLab for Proteomics” software. Using a peptide-centric approach, the software applied the binomial distribution to attribute a probability for each peptide as being linked to the disease; these probabilities were propagated to a final protein p-value, according to the Stouffer’s Z-score method. (TIF 195 kb) [file 12885_2019_5547_MOESM6_ESM.tif]
